# Dengue infection with multiorgan dysfunction:-sofa score, arterial lactate and serum albumin levels are predictors of outcome

**DOI:** 10.1186/2197-425X-3-S1-A830

**Published:** 2015-10-01

**Authors:** S Jog, S Prayag, P Rajhans, K Zirpe, S Dixit, L Pillai, J Shah, M Penurkar, A Kakrani, A Yadav, K Kadapatti, B Pawar, P Joshi, D Salunke, A Deshpande, D Patel, ISCCM Pune Research Initiative ( IPRI)

**Affiliations:** Deenanath Mangeshkar Hospital, Intensive Care Medicine, Pune, India; Shree Medical Foundation, Pune, India; Deenanath Mangeshkar Hospital, Pune, India; Ruby Hall Clinic, Pune, India; Sanjeevan Hospital, Pune, India; Sant Dnyaneshwar Hospital, Pune, India; Bharati Veedyapeeth Hospital, Pune, India; Shashwat Group of Hospitals, Pune, India; D.Y. Patil Hospital, Pune, India; Niramay Hospital, Pune, India; Jehangir Hospital, Pune, India; Mai Mangeshkar Hospital, Pune, India; ISCCM Pune Branch, Pune, India; John Hunter Children's Hospital, Newcastle, Newcastle, Australia

## Introduction

Mortality in severe dengue infections is attributed to development of multiple organ dysfunctions. The manifestations of severe dengue are varied and unfortunately the exact morbidity and mortality in terms of organ dysfunction are not well studied in Indian context. We did a prospective multicentre observational study in fourteen tertiary care intensive care units of Pune city during July 2012 to December 2013. The aim was to estimate mortality in severe dengue infections with multiorgan organ dysfunction.

## Methods

Newly admitted patients in the Intensive Care Units with history, clinical assessment and initial laboratory investigations suggestive / confirming diagnosis of Dengue Infection and having multiorgan dysfunction on SOFA (Sequential organ failure assessment) score (Severe Dengue) were screened for the enrollment in the study. Screening period was defined as first 24 hours within intensive care admission. Inclusion criteria were: Adults > 18 years of age, confirmed diagnosis of Dengue Infection by IgM or NS-1 antigen or Dengue PCR positive and at least two documented Organ Dysfunction by SOFA criteria. Exclusion Criteria were: ICU admission or study enrollment after 5 days of first documented multi organ dysfunction, Pregnancy or Confounding diagnosis (concurrent Malaria /Leptospira /Ricketssia/ Bacterial sepsis). Data was collected on demographics, clinical and laboratory variables. Data on SOFA scores was collected on day 1 and day 2 of enrollment into study.

## Results

A total of 113 patients met all inclusion and exclusion criteria. 30 (26·55%) patients did not survive and remaining 83(73·45%) patients survived. Median time to death amongst those who did not survive was 5 (1·5-9) days. Univariable and multivariable Cox Proportional Hazard Risk model created using baseline data on SOFA score, pH, need for external respiratory support, serum albumin level and highest arterial lactate to predict mortality revealed that worse SOFA score at baseline, higher arterial lactate at baseline and low serum albumin levels as significant predictors.

Every mmol/L increase in arterial lactates, the risk of mortality increases by a factor 0f 1.27 and similarly for every 1 unit increase in SOFA score at baseline, mortality increased by a factor of 1·23 Thus presence of organ dysfunction and especially cardiovascular organ dysfunction as evident by high arterial lactates is associated with higher risk of mortality. Analysis of Delta SOFA also revealed worsening SOFA score during intensive care unit stay (SOFA score on day 1 versus SOFA score o n day 2 was associated with increasing mortality.

## Conclusions

Our study showed significantly high mortality in patients with severe Dengue Infection especially those with non hematological organ dysfunction. Our study also showed strong correlation of SOFA score and Delta SOFA score, low serum albumin and high arterial lactate with prediction of mortality.

## Funding

**ISCCM Pune Research Initiative**

**Table 1:** Table 1

	Survivors (n = 83)	Non-survivors (n = 30)	Univariable Hazards ratio (95% C.I.))	p-value	Multivariable Hazards ratio (95% C.I.)	p-value
Highest Arterial lactate (mmol/L) Median (IQR)	1·7 (1·2 - 2·8)	7·7 (3·6 - 13·7)	1·26 (1·16 - 1·37)	< 0·001	1·27 (1·13 - 1·43)	< 0·001
Highest pH at baseline > 7.35 ≤ 7.35	54 (92%) 5 (8%)	14 (47%) 16 (53%	4·80 (2·29 - 10·04)	< 0·001	0·85 (0·23 - 3·17)	0·81
Mechanical ventilation No Yes	47 (60%) 31 (40%)	6 (20%) 24 (80%)	3.44 (1·40 - 8·43)	< 0·007	0·25 (0·06 - 1·09	0·07
Serum Albumin (gm/dL) > 3 ≤ 3	54 (67%) 27 (33%)	7 (23%) 23 (77%)	3·56 (1·51 - 8·39)	< 0·004	0·30 (0·09 - 0·97)	0·045
SOFA score on Day 1 Median (IQR)	5 (4-7)	11 (10-14)	1·25 (1·13 - 1·39)	< 0·001	1·23 (1·00 - 1·51)	0·05

British Journal of Haematology

**Table 2:** Table 2

	Alive	Died	Univariable HR 95% C.I.	p value	Multivariable HR 95 % C.I.	p value
SOFA Day 1 > SOFA Day 2	77 ( 93%)	3(10%)	19·05 (5·75 - 63·11)	< 0.001	14·85 (1·57 - 140·80)	0.01
SOFA Day 1 < SOFA Day 2	6(7%)	27 (90%)	19·05 (5·75 - 63·11)	< 0.001	14·85 (1·57 - 140·80)	0.01

*[Analysis of Delta SOFA]*

**Figure 1:**
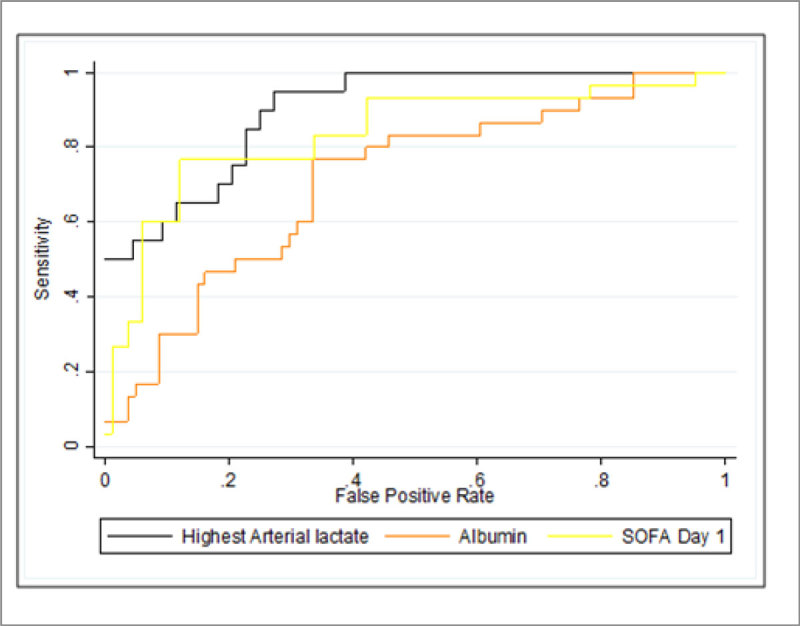
**ROC curve for mortality.**

